# Structural and Biochemical Characterization of SrcA, a Multi-Cargo Type III Secretion Chaperone in *Salmonella* Required for Pathogenic Association with a Host

**DOI:** 10.1371/journal.ppat.1000751

**Published:** 2010-02-05

**Authors:** Colin A. Cooper, Kun Zhang, Sara N. Andres, Yuan Fang, Natalia A. Kaniuk, Mandy Hannemann, John H. Brumell, Leonard J. Foster, Murray S. Junop, Brian K. Coombes

**Affiliations:** 1 Michael G. DeGroote Institute for Infectious Disease Research, Department of Biochemistry & Biomedical Sciences, McMaster University, Hamilton, Ontario, Canada; 2 Department of Biochemistry & Molecular Biology, University of British Columbia, Vancouver, British Columbia, Canada; 3 Program in Cell Biology, The Hospital for Sick Children, Toronto, Ontario, Canada; 4 Department of Molecular Genetics and the Institute of Medical Science, University of Toronto, Toronto, Ontario, Canada; University of California San Diego, United States of America

## Abstract

Many Gram-negative bacteria colonize and exploit host niches using a protein apparatus called a type III secretion system (T3SS) that translocates bacterial effector proteins into host cells where their functions are essential for pathogenesis. A suite of T3SS-associated chaperone proteins bind cargo in the bacterial cytosol, establishing protein interaction networks needed for effector translocation into host cells. In *Salmonella enterica* serovar Typhimurium, a T3SS encoded in a large genomic island (SPI-2) is required for intracellular infection, but the chaperone complement required for effector translocation by this system is not known. Using a reverse genetics approach, we identified a multi-cargo secretion chaperone that is functionally integrated with the SPI-2-encoded T3SS and required for systemic infection in mice. Crystallographic analysis of SrcA at a resolution of 2.5 Å revealed a dimer similar to the CesT chaperone from enteropathogenic *E. coli* but lacking a 17-amino acid extension at the carboxyl terminus. Further biochemical and quantitative proteomics data revealed three protein interactions with SrcA, including two effector cargos (SseL and PipB2) and the type III-associated ATPase, SsaN, that increases the efficiency of effector translocation. Using competitive infections in mice we show that SrcA increases bacterial fitness during host infection, highlighting the *in vivo* importance of effector chaperones for the SPI-2 T3SS.

## Author Summary

Systemic typhoid fever caused by *Salmonella enterica* serovar Typhi leads to high mortality in the developing world and can be linked with chronic, persistent infections in survivors. To cause disease, *Salmonella* uses a specialized secretion device called a type III secretion system to disarm cells of the immune system and replicate within them. The assembly and function of this secretion system requires a set of chaperone proteins to direct the process, but the chaperone proteins themselves have remained elusive. Here, we found a new chaperone protein, called SrcA, which is required for proper function of the type III secretion system. Using a bacterial mutant lacking the *srcA* gene, we found that this chaperone was needed for *Salmonella* to compete against wild type cells during systemic disease because it controls secretion of at least 2 key proteins involved in immune escape and cell-to-cell transmission. This chaperone is present in all types of virulent *Salmonella*, but not in *Salmonella* that don't cause human infections, providing new insights into the pathogenic nature of this organism.

## Introduction

Many Gram-negative bacteria that colonize host animals use a type III secretion system (T3SS) to deliver effector proteins directly into host cells where their interaction with host proteins and membranes contribute to pathogenesis. Comprised of over 20 proteins, T3SS are complex structures with relation to the flagellar T3SS [1],[2] and include several central features; (i) inner and outer membrane ring structures, (ii) an extracellular needle structure with pore-forming proteins at the distal tip that engage a host cell membrane, (iii) an ATPase at the base of the apparatus with energetic and chaperone-effector recruitment roles, and (iv) a suite of chaperones to coordinate the assembly and function of the apparatus during infection.

Secretion chaperones are proteins required for T3SS function with roles in apparatus assembly and effector delivery, but are not themselves subject to secretion [3]. These chaperones often have common physical features such as low molecular weight (<15 kDa), an acidic isoelectric point and a predicted amphipathic helix at the carboxyl terminus. Current literature groups secretion chaperones into three classes based on their physical interactions with cargo [3],[4]. Class I chaperones bind to translocated effector proteins at a chaperone binding domain (CBD) located in the amino terminus of the effector. Class I chaperones have a structural fold of five β-strands and three α-helices, forming homodimers that bind to the CBD in a horseshoe-like structure. These chaperones have been further sub-classified based on their substrate repertoire and location with respect to the genes encoding the T3SS [3]. Class II chaperones bind to translocon proteins that make up the secretion pore in the host target membrane and class III chaperones bind the extracellular filament proteins (or flagellin rod in the orthologous flagellar system) that polymerize into a helical structure following secretion from the bacterial cell. Secondary structure predictions suggest class III chaperones adopt an extended alpha helical structure, which was confirmed by the crystal structure of the CesA chaperone in enteropathogenic *E. coli* that binds the EspA filament protein [5].

Much of the virulence potential of *Salmonella enterica*, a group of more than 2300 serotypes, is attributed to horizontally acquired genomic islands termed *Salmonella* Pathogenicity Islands (SPI). SPI-1 encodes a T3SS required for host cell invasion and SPI-2 encodes a second T3SS needed for intracellular survival and immune evasion [6],[7]. To date, 13 effectors have been identified as substrates of the SPI-1 T3SS and 21 effectors for the SPI-2 T3SS, although the chaperones orchestrating the latter system have been elusive. Whereas 80% of the effectors of the SPI-1 system have defined chaperones, only two effector-chaperone interactions are known for the SPI-2 system. These include the effector-chaperone pair of SseF-SscB, and the chaperone SseA that binds translocon components SseD and SseB [8],[9],[10]. Crystal structures have been determined for three chaperones that coordinate translocation of effectors through the SPI-1 T3SS (InvB [11], SicP [12] and SigE [13]). However no structures are available for the SPI-2 T3SS chaperones whose effector repertoire seems considerably larger than that of the SPI-1 system.

In addition to maintaining a region of localized effector unfolding [12], T3SS chaperones have an emerging role as escorts that deliver their cargo to the cytoplasmic face of the inner membrane through physical interactions with an ATPase. These ATPases form a hexameric structure at the base of the T3SS [14] and are a conserved feature of both flagellar and non-flagellar type III systems to enhance secretion activity by promoting chaperone release and effector unfolding prior to secretion [15],[16],[17]. A chaperone-ATPase interaction for the SPI-2 T3SS has not been described previously and so whether this system conforms to the emerging escort paradigm is not known.

The regulation of the SPI-2 T3SS and its associated effector genes is coordinated by environmental cues signifying the intracellular environment [18]. These cues activate a two-component signaling system encoded in the SPI-2 island comprising the SsrA sensor kinase and SsrB response regulator. In addition to activating all of the T3SS structural operons, transcriptional profiling has uncovered new genes in the SsrB regulon that are required for bacterial pathogenesis including a translocated effector, SseL [19],[20], and a gene of unknown function called *srfN* that is common to the *Salmonella* genus [21]. Using a reverse genetics approach we identified an SsrB-regulated gene (STM2138) that we named *srcA* (SsrB-regulated chaperone A), whose gene product satisfied several a priori predictions relating to the physical properties associated with T3SS chaperones. We solved the crystal structure of SrcA and performed additional biochemical, proteomic and *in vivo* experiments that revealed SrcA to be a class I chaperone required for bacterial fitness in the host environment. Despite being genetically disconnected from SPI-2, SrcA is integrated functionally with this system by binding to the T3SS ATPase, SsaN, and providing chaperone activity towards two important effectors, SseL (*STM2287*) and PipB2 (*STM2780*), necessary for immune escape and cell-to-cell transmission. These data reveal structural and biochemical insight into a T3SS secretion chaperone required for intracellular pathogenesis of *Salmonella*.

## Results

### Identification of an SsrB-regulated secretion chaperone

**Figure 1:**
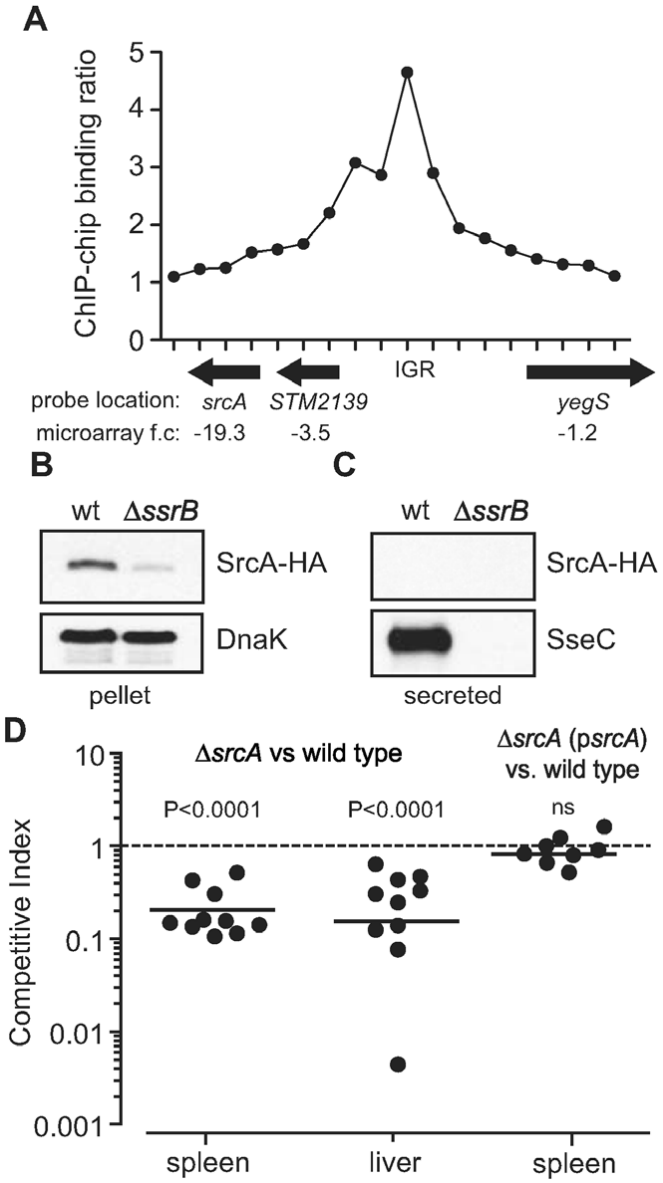
Identification of an SsrB-regulated gene, *srcA*. (**A**) Chip-on-chip analysis of the genomic region surrounding *srcA* including the 5′ upstream region. *In vivo* binding of SsrB was positive for 10 syntenic probes in the intergenic region (IGR) upstream of *srcA*. Each data point is a unique probe with averaged data from three biological replicates. Direction of gene transcription is shown by arrows beneath the abscissa and includes the fold-change in mRNA levels in *ssrB* mutant cells. (**B**) Accumulation of SrcA protein in cells requires SsrB. Cells harboring an *srcA-HA* allele were grown under SsrB-activating conditions and whole cell lysates (pellet) were probed by western blot for SrcA-HA, which accumulated in wild type cells (wt) but not in *ssrB* mutant cells (Δ*ssrB*). (**C**) SrcA is not secreted into the culture supernatant. Cell-free secreted protein fractions from wt and Δ*srcA* cultures were probed for SrcA-HA. SrcA-HA was not found in the secreted protein fraction from any of the cultures, whereas the type III-secreted protein, SseC, was secreted into the medium in an SsrB-dependent manner. (**D**) SrcA is required for competitive fitness *in vivo*. The ability of *srcA* mutant cells to compete with wild type parent cells was quantified by competitive infections of mice. Mice were infected orally with an equal proportion of mutant and wild type cells and the competitive index in the spleen and liver was determined at three days after infection. Complementation of *srcA* in trans (p*srcA*) restored the ability of *srcA* mutants to compete equally with wild type cells. Each data point represents an individual animal.

Transcriptional profiling of SsrB-regulated genes in *S. enterica* serovar Typhimurium (*S.* Typhimurium) [22] identified a hypothetical gene, *STM2138* (named *srcA* hereafter), that was co-regulated with genes in SPI-2 and repressed ∼20-fold in an *ssrB* mutant compared to wild type. This gene was also down regulated in *Salmonella* mutants lacking the SsrA sensor kinase [20], and was predicted to encode a possible chaperone in a bioinformatics-based screen [23]. The *srcA* gene is not located in the vicinity of the T3SS encoded by SPI-2 (*STM1378-STM1425*), but is 713 genes downstream on the chromosome (STM numbers are based on the LT2 genome and ordered sequentially on the chromosome beginning at STM0001, *thrL*). The predicted *srcA g*ene product was a small protein ∼16 kDa with a pI of 4.6, similar to secretion chaperones associated with T3SS. To verify SsrB input on *srcA* expression, we analyzed SsrB binding *in vivo* at the region of DNA surrounding *srcA* using genome-wide ChIP-on-chip [21] (and unpublished data). This analysis revealed a strong SsrB binding site spanning 10 syntenic probes within the intergenic region (IGR) upstream of *srcA*, that together with the transcriptional data corroborated a direct regulatory role for SsrB on *srcA* expression (**Fig. 1A**). To determine the cellular distribution of SrcA we constructed a *srcA-HA* allele and expressed this gene in wild type and in *ssrB* mutant cells under conditions that activate the SPI-2 T3SS [24]. In whole cell lysates, SrcA protein was reduced ∼10-fold in Δ*ssrB* cells compared to wild type (**Fig. 1B**) and the protein was not detected in the secreted fraction from wild type cells (**Fig. 1C**), consistent with the expected properties of a T3SS chaperone. As a positive control, SseC, an SsrB-regulated translocon protein of the SPI-2 T3SS was present in the secreted fraction from wild type cells but not from an *ssrB* mutant.

### SrcA contributes to *Salmonella* fitness in an animal host

Most SsrB-regulated gene products contribute to the intracellular survival of *Salmonella* in a host. In comparative genomics analyses, *srcA* was found in all virulent strains of *Salmonella enterica* containing SPI-2, but was absent from the cold-blooded animal commensal, *S. bongori*, which lacks SPI-2 (**Table S1**). This suggested a co-evolution of *srcA* with the SPI-2 T3SS and a possible functional relationship. If so, we reasoned that SrcA should contribute to animal colonization because the SPI-2 T3SS is essential for host infection. To determine whether SrcA contributes to *Salmonella* fitness in a host, we created an unmarked in-frame *srcA* deletion in *S.* Typhimurium and competed this strain against wild type cells in mixed oral infections of mice [25]. After three days of infection the geometric mean competitive index (CI) for the mutant was 0.20 (95%CI 0.13–0.29) and 0.18 (95%CI 0.06–0.5) in the spleen and liver respectively (*P*<0.0001; **Fig. 1D**) indicating that bacteria lacking *srcA* were significantly out competed by wild type cells during systemic infection. To verify the role of *srcA* on this phenotype, we complemented the *srcA* mutant with a wild type *srcA* gene under the control of its endogenous promoter, which restored *in vivo* fitness to that of wild type (**Fig. 1D**). The level of attenuation of the *srcA* mutant was generally higher than most single effector gene mutants [26], which suggested to us that SrcA contributes to an important aspect of T3SS function *in vivo*.

### Crystal structure of SrcA

**Figure 2:**
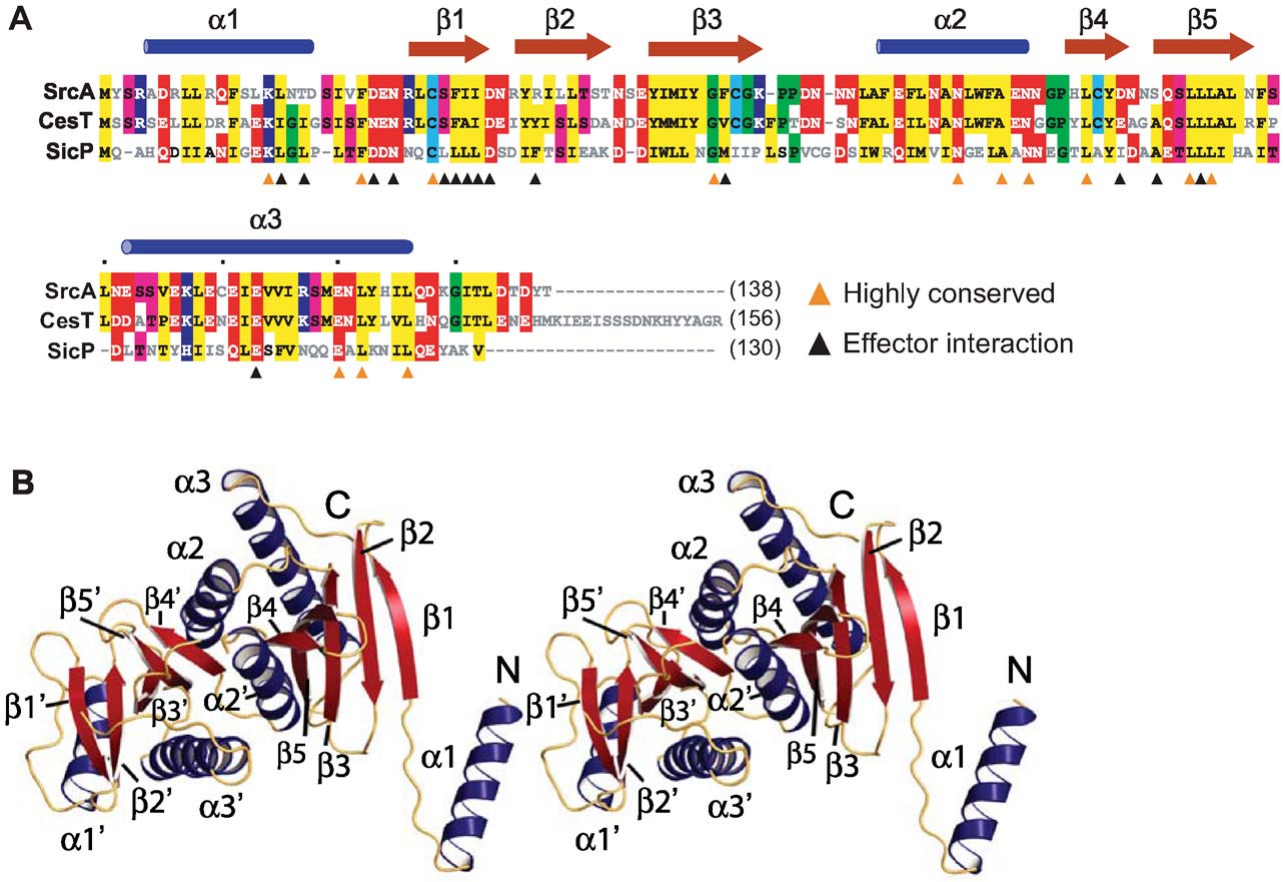
Structural characterization of SrcA. (**A**) Structure-based sequence alignment of full-length SrcA with structural orthologs CesT from enteropathogenic *E. coli* and SicP from *S.* Typhimurium. Orange arrowheads denote residues conserved between all three proteins; black arrowheads denote residues that participate in the cargo-binding interface as defined in [12] (PDB 1JY0). Conserved residues are colored as follows: hydrophobic, yellow; negative charge, red; positive charge, blue; threonine, serine, pink; cysteine, light blue; proline, glycine, green. (**B**) Stereo image of a SrcA dimer with α-helices and β-strands in blue and red respectively.

Sequence analysis showed 59% amino acid identity between SrcA and CesT, a secretion chaperone in enteropathogenic *E. coli* (EPEC) (**Fig. 2A**). As a means to address the biological function of SrcA, we solved the crystal structure at 2.5-Å resolution (**PDB 3EPU**). A summary of crystallographic data collection and model refinement statistics is in **Table 1**. The structure was solved by molecular replacement using an initial model based on CesT (PDB 1K3E) [13]. SrcA crystallized in space group C2 with two molecules related by a 2-fold symmetry axis in each asymmetric unit (**Fig. 2B**). Each monomer consisted of a small and large domain. The smaller domain formed by α1 and the extended loop region preceding β1 adopts a distinct conformation in each subunit. The larger domain mediates dimerization and is comprised of a twisted anti-parallel β-sheet (β1-β2-β3-β5-β4) flanked by α-helices α2 and α3. The dimer interface formed between SrcA monomers occurs primarily through reciprocal hydrophobic interactions between α2 and α2′ with additional interface-stabilizing interactions occurring between the α2 helix of one monomer and β4 and β5 strands of the opposing monomer (**Fig. 2B**). The total surface area buried at the dimer interface is 1258 Å^2^, suggesting that SrcA would exist as a dimer in solution, which we confirmed by gel filtration analysis (see below).

**Table 1:** Crystallographic Data and Refinement Statistics.

**Data collection**	
Wavelength (Å)	1.1
Space group	*C*2
Cell parameters	a = 103.84, b = 46.83, c = 65.94
	α = γ = 90; β = 106.39
Molecules in A.U.	2
Resolution range (Å)^a^	50–2.49 (2.58–2.49)
Unique reflections	10664
Data Redundancy^a^	3.62 (3.58)
Completeness (%)^a^	98.5 (94.6)
I/σ(I)^a^	13.5 (3.4)
R_merge_(%)^a^	6.4 (31.6)
Wilson scaling *B* factor (Å^2^)	54.8
**Model and refinement**	
Resolution range (Å)	50–2.5
R_work_ (%)	21.45
R_free_ (%)	25.26
Refl. observed	10170
Refl. test set	538
No. of protein atoms	2174
No. of waters	86
rmsd bond lengths (Å)	0.012
rmsd bond angles (Å)	1.371
Average *B* factor (Å^2^)	63.8

a Data for the highest resolution shell are shown in parentheses.

A structure similarity search with SrcA revealed proteins identified as T3SS secretion chaperones. CesT and SicP were the most structurally similar to SrcA, aligning with RMSD of 1.8 Å and 2.2 Å respectively. With the exception of CesT, SrcA has weak overall sequence identity (<20%) with other T3SS chaperones. CesT, SicP and SrcA contain several clusters of highly conserved amino acids notable on primary sequence alignments (**Fig. 2A**). Most of these conserved sites are located in the α2-interface helix and in strands β4 and β5 that help stabilize this interface. Although the N-terminus of these proteins is conserved structurally, the tertiary structures differ for each protein. In CesT, α1 and β1 adopt an extended conformation while the equivalent domain in SicP remains closely packed against the dimerization domain [12]. In SrcA, both extended and closely packed conformations are observed in separate subunits of the same dimer within the asymmetric unit. In the extended conformation the N-terminal helix from one dimer interacts with the β4 region of an adjacent dimer, similar to a domain swap seen in CesT [13]. At this time, the possible biological relevance for such a domain swap is unclear and may reflect an artifact of crystallization as critically discussed [13].

**Figure 3:**
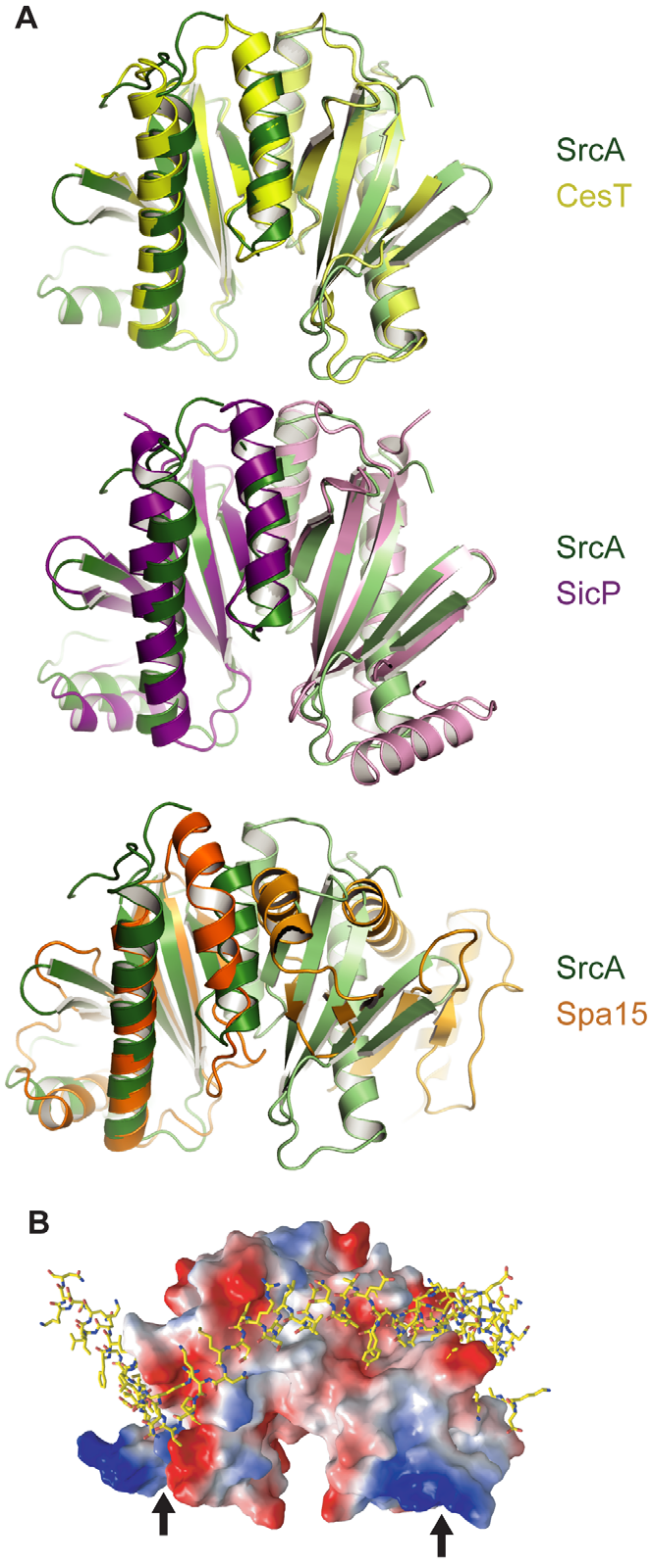
Structural comparison of SrcA, CesT and SicP. (**A**) Crystal structures of SrcA, CesT, and SicP dimers highlighting the dimer interface. The two monomers of each chaperone dimer are colored in light and dark colors (green, SrcA; yellow, CesT; purple, SicP; orange, Spa15). (**B**) Space-filling model of SrcA bound to the SicP effector, SptP, based on a structural alignment. Negatively charged patches are red, positively charged patches are blue, and hydrophobic patches are white. Black arrows indicate the N-terminus.

A comparison of the SrcA dimer interface with other class I chaperone family members indicates the overall similarity of quaternary structure shared between SrcA, CesT and SicP (**Fig. 3A**). This is in contrast to the class II chaperone interface of Spa15, which despite having similar tertiary structure to SrcA adopts a distinct dimer interface. A structural alignment of SrcA and Spa15 generated through alignment of single monomers shows the relative difference in subunit orientation between SrcA and Spa15 reflected by the positions of each monomer in the dimer configuration. These unique orientations produce an 80° rotational offset between respective subunits and could be expected to influence the mode of effector interactions utilized by these proteins.

To evaluate the potential for an effector-binding surface on SrcA, the structure of SicP in complex with its effector SptP was aligned with SrcA and represented as a space-filling model (**Fig. 3B**). Binding of SptP occurs primarily in the N-terminus of SicP [12], which is similar to the effector binding surface for SrcA predicted *in silico*. This surface contains several conserved hydrophobic residues including L16, D24, N26, and I32 (**Fig. 2A**), which is consistent with SrcA using a similar mechanism for interaction with effectors.

### SrcA interacts with the SPI-2 T3SS ATPase

**Figure 4:**
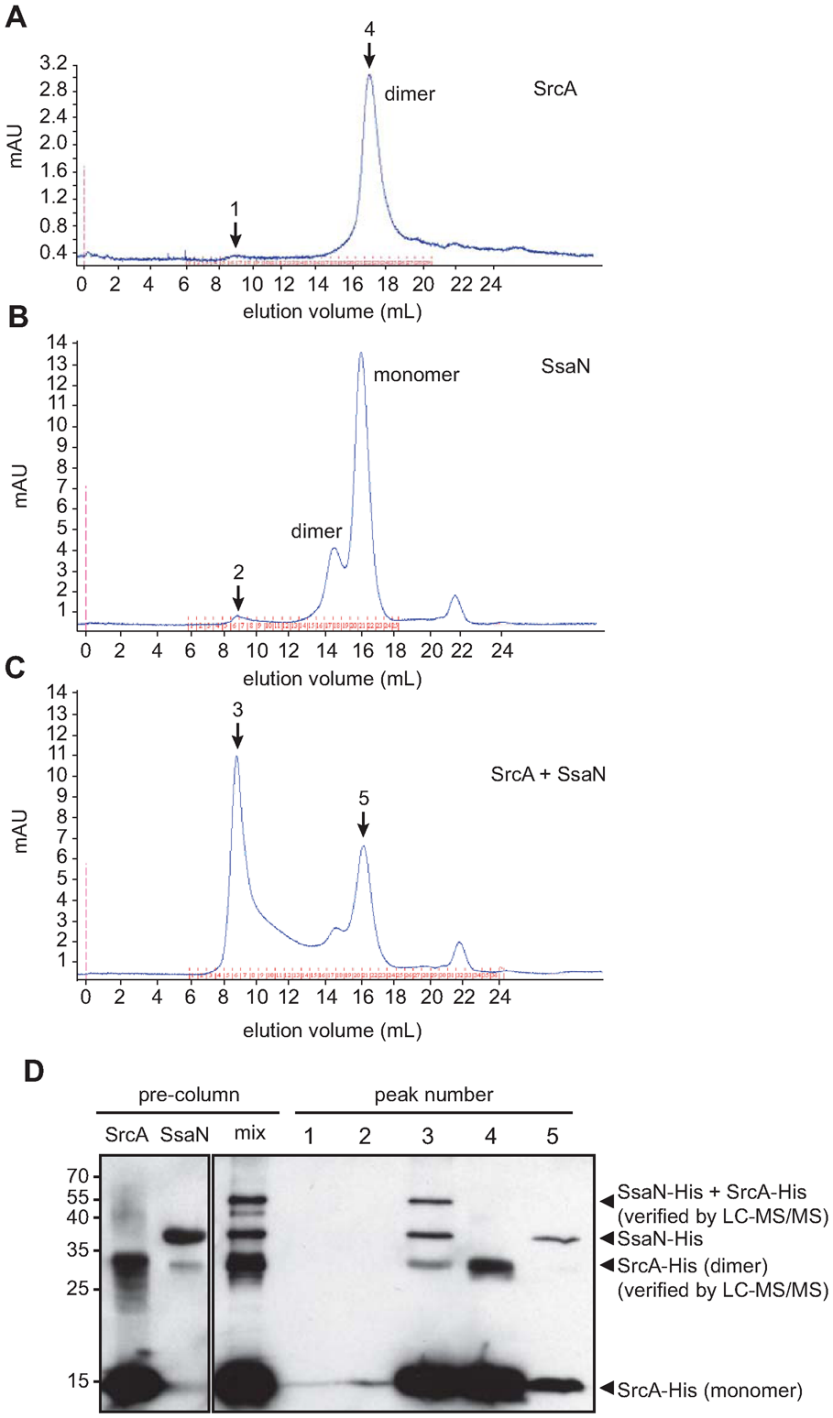
SrcA binds to the type III ATPase, SsaN, and induces its multimerization. Gel filtration chromatography was used to analyze (**A**) purified SrcA, (**B**) purified SsaN, and (**C**) a mixture of SrcA with SsaN. (**D**) Peak fractions (black arrows) in the elution volumes were collected and analyzed by western blot with an anti-His antibody to identify protein compositions. Pre-column samples of SrcA migrated as monomer and dimer species in SDS-PAGE gels (verified by LC-MS/MS) while SsaN was prominently a monomer. Incubation of SsaN with SrcA chaperone induced the formation of a high-molecular weight oligomeric species comprised of both proteins, which was only present in SsaN-SrcA mixtures.

An emerging function for T3SS chaperones is delivery of cargo to the base of the apparatus through interactions with an ATPase. This was shown for the flagellar T3SS [17] and later in the virulence-associated T3SS in *E. coli* [16],[27] and the SPI-1 T3SS in *Salmonella* [15]. However, analogous interactions have not been described for the SPI-2 T3SS. Since *srcA* expression was co-regulated with genes in SPI-2, we hypothesized that it had a functional role in this system. To address this biochemically we purified SrcA and the predicted ATPase for the SPI-2 T3SS, SsaN, and performed binding experiments and gel filtration chromatography of the protein mixtures. SsaN contains conserved amino acid residues characteristic of Walker-A and Walker-B motifs of P-loop nucleoside triphosphate hydrolases, as well as a number of residues shown to contribute to ATP binding or ring stacking with the adenine base of ATP in the *E. coli* orthologue, EscN, (Q412, E191, R366) (**Fig. S1**). Since SsaN had not been characterized biochemically we first verified that our purified protein had ATPase activity (**Fig. S1**). We then mixed SrcA and SsaN proteins and resolved the protein complexes by gel filtration chromatography. By itself, SrcA existed as a dimer in solution (**Fig. 4A**) with no higher oligomers present, substantiating the stoichiometry obtained from our crystal data. SsaN existed as a monomer with a minor population eluting in a volume consistent with a probable dimer (**Fig. 4B**). When SrcA was mixed with SsaN, a new protein complex of high molecular weight was observed, along with diminished peaks corresponding to the SrcA dimer and SsaN monomer (**Fig. 4C**). This new complex elutes with a Stokes radius consistent with an apparent molecular mass of ∼600 kDa. We verified the identities of protein originating from each peak by western blot (**Fig. 4D**) and LC-MS/MS, which showed the new complex was comprised of both SsaN and SrcA.

### SrcA binds effector cargo destined for the SPI-2 T3SS

**Figure 5:**
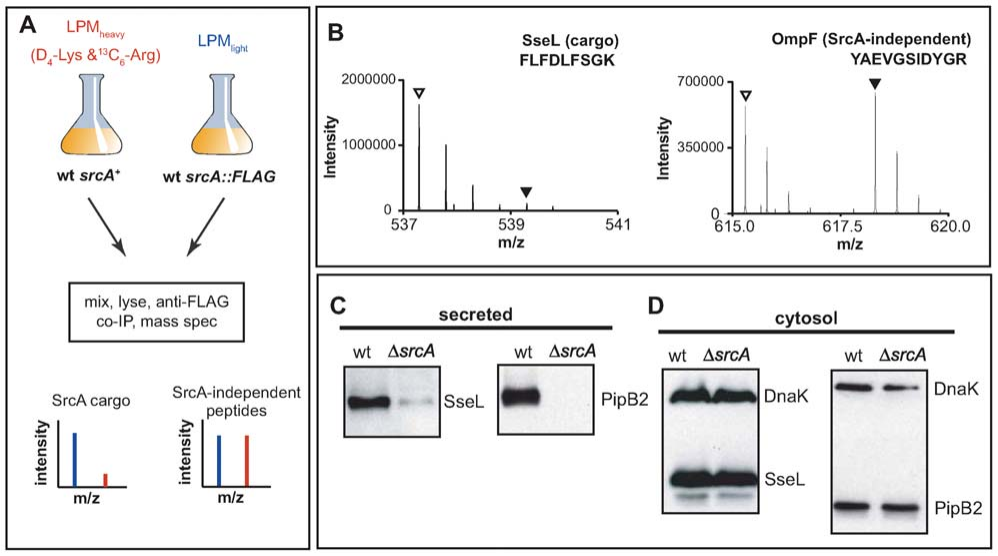
SrcA is a multi-cargo secretion chaperone for SseL and PipB2. (**A**) SILAC was used in conjunction with quantitative mass spectrometry-based proteomics for systematic identification of effector cargos bound by SrcA *in vivo*. (**B**) Mass:charge spectra for representative peptides (sequences shown) identified from SseL and OmpF, where normal isotope abundance-labeled peptides are indicated with open triangles and ^2^H_4_-Lys or ^13^C_6_-Arg labeled peptides are indicated by filled triangles. Protein abundance of SseL and PipB2 was examined by western blot in wild type and Δ*srcA* mutant cells from secreted (**C**) and cytosolic protein fractions (**D**). Data is representative of 4 to 6 experiments.

Since structural and biochemical data unambiguously defined SrcA as a T3SS-associated chaperone, we used two experimental approaches to identify SrcA cargo(s). First, we used stable isotope labeling of amino acids in cell culture (SILAC) [28] in conjunction with quantitative mass spectrometry-based proteomics to identify cargo immunoprecipitated with SrcA from *Salmonella*. For this series of experiments we constructed a mutant in which the *srcA* gene was replaced on the chromosome with *srcA-FLAG* to enable immunoprecipitation from cell lysates. Lysates prepared from wild type cells grown in ^2^H_4_-Lys and ^13^C_6_-Arg containing SILAC medium (heavy) and *srcA* mutant cells grown in medium containing natural amino acids of Lys and Arg (light) were mixed and subjected to an immunoprecipitation procedure with an anti-FLAG antibody followed by quantitative mass spectrometry. Peptides originating from wild type cells contained heavy atom-substituted lysine and arginine such that putative SrcA cargo proteins would generate low heavy:light SILAC peptide ratios from the complex mixtures (**Fig. 5A**). In these experiments the T3SS effector protein SseL was identified by quantitative SILAC mass spectrometry as a specific SrcA cargo protein (**Fig. 5B**). SseL was immunoprecipitated specifically along with SrcA-FLAG with a SILAC ratio of 0.08, whereas additional abundant proteins displayed SILAC ratios closer to ∼1 (OmpF is shown, **Fig. 5B**) (mean SILAC ratio of all other peptides identified was 0.93 (**Dataset S1**).

Secondly, to verify the mass spectrometry data and to identify other possible effector cargo, we examined the secretion profiles of wild type cells and an *srcA* mutant that each expressed HA-tagged effector genes, the products of which are secreted by the SPI-2-encoded T3SS. Using this approach SseL-HA and PipB2-HA were depleted from the secreted protein fraction of *srcA* mutant cells (**Fig. 5C**) but reached similar levels in the bacterial cytoplasm (**Fig. 5D**). As expected from data with the complemented mutant *in vivo*, expression of *srcA* in trans restored effector secretion in the *srcA* mutant (data not shown).

### SrcA is required for PipB2-dependent centrifugal displacement of the *Salmonella*-containing vacuole

**Figure 6:**
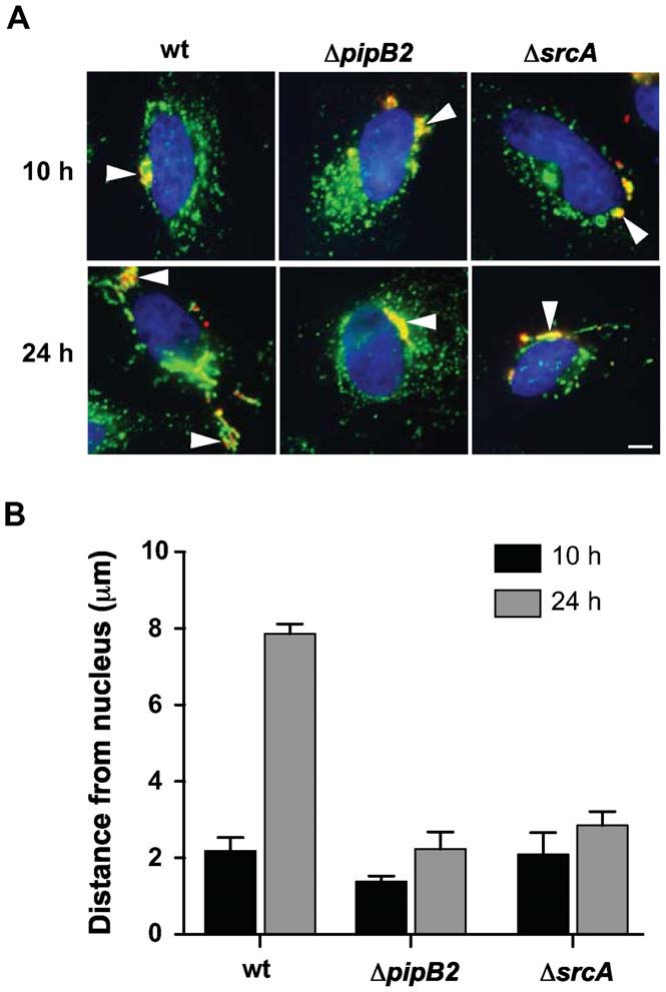
SrcA is required for centrifugal movement of the SCV. (**A**) Intracellular positioning of SCVs containing wild type *Salmonella* or Δ*pipB2* and Δ*srcA* mutants after 10 and 24 h post-infection. HeLa cells were immunostained for bacteria (red) and LAMP1 (green), and the nucleus stained using DAPI (blue). Arrowheads indicate SCVs. Bar represents 10 µm. (**B**) HeLa cells were infected with wild type or Δ*pipB2* and Δ*srcA* mutants of *S.* Typhimurium. Cells were fixed at the indicated times, immunostained and analyzed as described in Methods. Means with average deviation for two separate experiments are shown.

To further show a role for SrcA in chaperoning PipB2, we set up experiments to test whether deleting *srcA* would phenocopy Δ*pipB2* cells for PipB2-dependent centrifugal displacement of the *Salmonella* containing vacuole (SCV) in epithelial cells, an event linked to cell-to-cell transfer during infection *in vitro* [29]. At 10 h after infection the majority of SCVs were situated near the nucleus in accordance with previous work (**Fig. 6A**) [29]. By 24 h after infection SCVs containing wild type bacteria were displaced centrifugally towards the cell periphery whereas SCVs containing either *pipB2* or *srcA* mutant bacteria remained juxtaposed to the nucleus (**Fig. 6A**). The average distance from the nucleus of LAMP1+ SCVs containing wild type bacteria was 2.19 µm at 10h post infection and increased to 7.86 µm by 24 h after infection. Conversely, SCVs containing either Δ*pipB2* cells or Δ*srcA* cells were 1.38 µm and 2.09 µm at 10h but lacked centrifugal displacement at 24 h (2.23 µm and 2.85 µm, respectively) (**Fig. 6B**).

## Discussion

### Structural features of SrcA

We used a reverse genetics approach to define a new secretion chaperone in *S.* Typhimurium that is integrated functionally with the T3SS encoded by SPI-2, a system well described for its role in immune subversion and intracellular infection during host colonization. Consistent with other class I secretion chaperones, SrcA has extensive electronegative charge distributed over the surface of the molecule. The exact function of this charge distribution is not known, but data from other systems suggests a docking recognition function with other components of the type III apparatus, possibly the T3SS-accociated ATPase. For instance, electronegative surface residues on the SigE chaperone in the SPI-1-encoded T3SS negatively affect cargo secretion, but not cargo stability [30]. In enteropathogenic *E. coli*, a surface-exposed electronegative residue in the CesT chaperone (Glu142) likewise contributes to Tir secretion but not Tir binding [16], suggesting a role in either targeting bound cargo to the T3SS or in the secretion process itself. Interestingly, SrcA lacks 17-amino acids that make up the carboxyl terminus of CesT, which includes Glu142, and yet it still retains effector binding, ATPase binding and effector secretion functionalities. Thus, it is likely that other surface charged residues of SrcA are involved in these functions or that SrcA targets effector cargo to the secretion apparatus through a mechanism distinct from CesT.

The interface for the SrcA homodimer is extensive and is more in keeping with the structural features of single-effector class IA chaperones (∼1100–1300 Å^2^) compared to the reduced dimer interface of Spa15, a multi-cargo class IB chaperone from *Shigella* [31]. Similar to CesT and SicP, the dimer interface of SrcA adopts a parallel configuration when comparing α2 helices of opposing subunits. In contrast, the subunits of Spa15 undergo a significant relative rotation (80°) about the α2-axis resulting in a different interface. These features may relate to biological function in the SPI2 T3SS and/or in vetting effector cargo amongst the >30 effectors identified in *Salmonella*. We found no evidence of interactions between SrcA and translocon components of the SPI-2 T3SS and so it appears as though SrcA functions specifically in effector translocation events.

### Implications for type III secretion function

The interaction between SrcA and SsaN supports an emerging paradigm whereby secretion chaperones bring effector cargo to the T3SS through physical interaction with the hexameric ATPase at the base of the apparatus [14]. This was demonstrated for chaperone-ATPase components in the flagellar type III system [17] and in non-flagellar type III systems in *E. coli* [27] and the SPI-1 system in *Salmonella* [15]. Our work shows the first chaperone-ATPase interaction for a T3SS functioning from within an intracellular vacuolar compartment and supports this interaction as a more generalize feature of type III secretion function. In our experiments, we could induce the ATPase domain of SsaN to oligomerize in the presence of SrcA, but not in its absence, which was intriguing because the purified enzyme lacked a domain at the carboxyl terminus thought to be involved in oligomer stability, at least for *E. coli* EscN [14]. These data suggest that type III chaperones might have an as yet undefined role in assembly of the ATPase homohexamer that gives rise to efficient effector translocation. This will be an important area for further experimentation in this and other systems.

### Genetic and functional integration of SrcA with type III secretion

The genes encoding the *srcA* chaperone and the effector cargos (*pipB2* and *sseL*) are found in all serotypes of *Salmonella enterica* that contain the SPI-2-encoded T3SS. Conversely, these genes are absent from *S. bongori*, which lacks the SPI-2-encoded T3SS. The expression of *srcA* is coordinated with T3SS transcriptional activity via the SsrA-SsrB two-component regulatory system encoded in SPI-2. The direct binding of SsrB to the promoter region upstream of *srcA*, along with SsrB-regulation of both *sseL* [19],[20] and *pipB2* [32] is indicative of multiple *cis*-regulatory mutation events that have allowed for functional coordination of the distributed secretion apparatus, chaperone and effector cargos. We recently described this type of regulatory evolution for pathogenic adaptation of *Salmonella* to its host [21] and *srcA* is consistent with regulatory evolution of chaperone-effector gene pairs that are not co-transcribed in operons.

Due to low G+C base content compared to the genome average of 52%, it's likely that *srcA* (32% G+C) and an adjacent gene, *STM2137* (37% G+C), were acquired as a foreign islet that was retained in organisms containing the SPI-2 T3SS due to the selective advantage afforded by the new protein interactions so created. Interestingly, STM2137 (also known as SseK2) is a likely paralog of SseK1, an effector translocated by the SPI-2 T3SS [33]. SseK2 is also regulated by the SsrA-SsrB two-component system but compared to SseK1, it is translocated in much less abundance into host cells [33]. Using the methods described here, we were not able to detect SseK2 secretion or a physical interaction with SrcA, however it remains possible that SrcA also chaperones SseK2 for low-level translocation.

SrcA is unique among other multi-effector chaperones most closely related to it in that it is unlinked from the T3SS genomic island. For example, InvB and SicP (*Salmonella* SPI-1), CesT (enteropathogenic *E. coli* locus of enterocyte effacement) and Spa15 (*Shigella mxi/spa* virulence plasmid region) chaperones are all encoded within the T3SS structural operons, implying they have co-evolved as a single genetic entity from a common ancestor. Given its genetic neighborhood, *srcA* appears to be a genetic acquisition separate from SPI-2 that functionally links some effectors to the T3SS apparatus via the ATPase. The role of horizontal gene transfer and regulatory evolution in allowing for uncoupling of chaperones, effectors and the T3SS has many possible implications for T3SS function, including plasticity in chaperone-effector interaction networks, expansion of effector repertoires, and alterations to the kinetics and hierarchical delivery of effectors to a host cell. These events may improve host adaptability or even expand the host range of bacteria that acquire and integrate new functional secretion chaperones.

## Methods

### Ethics statement

All experiments with animals were conducted according to guidelines set by the Canadian Council on Animal Care. The local animal ethics committee, the Animal Review Ethics Board at McMaster University, approved all protocols developed for this work.

### Bacterial strains and growth conditions

*Salmonella enterica* serovar Typhimurium strain SL1344 was used as the wild type strain and all mutants were isogenic derivatives. Chromosomal replacements were done using a λ-Red-based technique described previously [34]. A synthetic minimal medium for isotopic labeling of proteins in cell culture was developed for SILAC proteomics experiments based on LPM medium that activates the SsrA-SsrB two-component regulatory system for induction of SsrB-regulated genes [24]. LPM medium was modified for compatibility with quantitative SILAC mass spectrometry by replacing casamino acids with individual l-amino acids and containing either natural l-arginine and l-lysine, or ^13^C-subsituted arginine (^13^C_6_-Arg) and deuterium-substituted lysine (^2^H_4,4,5,5_-Lys) (Cambridge Isotope Laboratories, Andover, MA). A full description of LPM-SILAC medium is provided in **Protocol S1**.

### Protein production and purification

For purification of His-tagged SrcA, the *srcA* gene was amplified from *S.* Typhimurium chromosomal DNA and cloned into pET-3(a) (Novagen) as a C-terminal fusion to a 6-histidine tag. Expression plasmids were transformed into *E. coli* Rosetta (DE3) and cells were grown in 1-L LB broth and induced with IPTG at OD_600nm_ 0.6 for 3 h at 37°C. Harvested cells were resuspended in 25 ml NiA buffer (20 mM Tris pH 8.5, 500 mM KCl, 20 mM imidazole, 0.03% LDAO and 10% glycerol), lysed using a French press and centrifuged at 48,383 *g* for 40 min. Soluble His-tagged protein was purified using nickel-chelating resin (GE Healthcare Life Sciences), followed by Mono-Q anion exchange using a 20 mM Tris pH 7.5, 500 mM KCl, 10% glycerol. Purified protein was exchanged into a final buffer of 20 mM Tris pH 7.5, 100 mM KCl, 10% glycerol and concentrated to ∼5 mg/mL. All SrcA purification steps were carried out at room temperature. For purification of His-tagged SsaN, a soluble protein form containing the ATPase domain and C-terminal domain spanning residues Q90-E433 was constructed according to previous work done on *E. coli* EscN [14]). SsaNΔ89 was purified from *E. coli* Rosetta (DE3) cells containing a pET-3(a) plasmid with the *ssaNΔ89* gene. Cells were sub-cultured into 1-L of Terrific Broth (TB) and grown with shaking at 200 rpm at 20°C for 65 h for auto-induction. Cells were harvested and lysed using a French press and soluble protein was purified using nickel chromatography and ion-exchange chromatography as described above. Finally, SsaN protein was concentrated to ∼9 mg/ml. All purification steps for SsaN were carried out at 4°C.

### Crystallization, data collection and structure determination

Crystals were generated via the hanging drop method by vapor diffusion using purified protein combined with crystallization solution (100 mM Bis-Tris propane pH 7.0, 200 mM MgCl_2_, 35% PEG 3350, 3.95 mM FOS-choline-9, 5% Jeffamine M-600) at a 3.5∶1 ratio, and equilibrated over 500 µL of 1.7 M ammonium sulfate, at 298K. After initial crystals were formed, drops were moved over wells containing 500 µL of 3 M ammonium sulfate and further equilibrated for 2 to 3 weeks. A single native data set, collected to 2.5 Å at the National Synchrotron Light Source Beamline X12C (Brookhaven, NY) was processed using HKL2000. An initial structural solution was achieved using molecular replacement with the type III chaperone CesT (PDB ID 1K3E) as the starting search model. PHENIX was used for model building [35]. Further model building and refinement was conducted iteratively using COOT and REFMAC [36],[37]. The final structure had R and R_free_ values of 21.4 and 25.3 respectively.

### Competitive infection of mice

For competitive infections, female C57BL/6 mice (Charles River) were infected per os with a 1∶1 mixed inoculum containing *srcA* mutant cells and a marked wild type strain resistant to chloramphenicol as described previously [21]. Competitive index (CI) was calculated in the liver and spleen at 3 days after infection as: cfu (mutant/wild type) _output_/(mutant/wild type) _input_. For complementation experiments, *srcA* was cloned with its native promoter into the low-copy plasmid pWSK29 and transformed into Δ*srcA* cells. The complemented mutant was competed in CI experiments against wild type cells transformed with empty pWSK29.

### Co-immunoprecipitation experiments and quantitative SILAC mass spectrometry

Co-immunoprecipitations were performed with M2-Agarose beads conjugated with anti-FLAG antibody (F-gel, Sigma, Oakville, ON). Wild type bacteria and bacteria with a *srcA-FLAG* allelic replacement were grown overnight in LB broth, washed in SILAC-LPM (Protocol S1), and sub-cultured 1∶50 into SILAC-LPM containing either ^12^C_6_-Arg and H_4_-Lys (light) or ^13^C_6_-Arg and ^2^H_4_-Lys (heavy) amino acids. Isotopic labeling of proteins was carried out until the culture reached an optical density of 0.6 at 600 nm. Cells were washed with PBS, pelleted at 3000 *g* for 10 minutes and resuspended in PBS containing mini-EDTA tablet (1 per 10 ml) protease inhibitors (PBS-PI) (Roche, Mississauga, ON). Cells were sonicated six times for 30 seconds each with 1 min intervals on ice (Misonix Sonicator 3000, Misonix, Farmingdale, NY). Samples were centrifuged at 3000 *g* for 15 minutes and the resulting supernatants from heavy and light samples were mixed. F-gel was equilibrated with PBS-PI containing 10 µg/ml BSA for 60 minutes and then lysates were immunoprecipitated with F-gel for 16 h at 4°C. F-gel was washed with PBS-PI ten times for 30 min each wash. Bound proteins were eluted with either FLAG peptide or twice with SDS-sample buffer (1 M Tris pH 8.0, 20% SDS, 0.5 M EDTA pH 8, 10% glycerol, 200 mM dithiothreitol). Final protein preparations were filter-concentrated, washed with water and diluted to a final concentration of 50 mM ammonium bicarbonate and 1% sodium deoxycholate. Proteins were digested in solution and analyzed by liquid chromatography-tandem mass spectrometry exactly as described previously [38].

### Gel filtration chromatography

Two hundred microlitres of purified SrcA protein in gel filtration buffer (20 mM Tris pH 7.5, 200 mM KCl; protein concentration, 1.1 mg/ml) was injected into a Superdex S200 10/300GL gel filtration column (Amersham Biosciences, Piscataway, NJ) at 0.2 ml/min. Elution fractions (0.5 ml) were collected at a flow rate of 0.5 ml/min. For SsaN, 40 µl (8.84 mg/ml) was diluted with 320 µl gel filtration buffer and injected into an S200 column as described. For mixing experiments, 320 µl of SrcA (0.1mg/ml) was mixed with 40.8 µl SsaN (8.84 mg/ml) at room temperature for 2 h. The mixture was centrifuged at 10,000 *g* for 5 minutes and the top two hundred microlitres of the supernatant was injected into an S200 column. Peak fractions were collected and protein identities in all peaks were verified by Western blot and LC-MS/MS.

### Type III secretion assays

Experiments to monitor secretion of type III effectors were performed according to previously published methods [19]. Wild type cells and an *srcA* mutant used for these experiments were transformed with low-copy plasmids expressing HA-tagged effector genes from their endogenous promoters (*sifA*, *sopD2*, *gogB*, *pipB*, *sseK2*) or contained allelic replacements on the chromosome to express HA-fusion proteins (*pipB2*, *sseL*). Antibodies used for Western blots were: mouse anti-HA (1∶1000), mouse anti-DnaK (1∶5000), rabbit anti-SseC (1∶20000). Secondary antibodies conjugated to horseradish peroxidase (HRP) were used at 1∶5000 and antigen-antibody complexes were detected using enhanced chemiluminescence (ECL).

### ATPase assay

ATPase activity of SsaN was measured using the pyruvate kinase-lactate dehydrogenase coupled assay that monitors NADH oxidation coupled with ATP hydrolysis [39]. Data was plotted as a decrease in absorbance at 340 nm over time.

### SCV positioning experiments

The intracellular position of *Salmonella*-containing vacuoles was determined by measuring the distance of LAMP1+ SCVs to the nearest edge of the host cell nucleus (labeled by DAPI staining) in fixed HeLa cells as previously reported [29]. Measurements were made using Openlab 3.1.7 software. Experiments were done in duplicate and the resulting finalized average was calculated from two independent average values (at least 100 measurements per experiment). Average distances with average deviation are reported.

### Coordinates

The coordinates and structure factors of SrcA have been deposited in the Protein Data Bank (accession code 3EPU).

## Supporting Information

Figure S1 Sequence and biochemical analysis of *Salmonella* SsaN. (A) Amino acid sequence alignment of SsaN and EscN from *E. coli*. The conserved Walker A and Walker B boxes from P-loop NTPases are shown in red and blue, respectively. Additional conserved catalytic residues are indicated in green including: Glu191 (conserved in position and orientation in EscN with F1 ATPase β-subunit); Arg352 (protrudes from adjacent monomer to bind ATP γ-phosphate in the ATP binding pocket of adjacent monomer); Gln412 (stabilizes ATP binding pocket in EscN). Residues correspond to SsaN numbering. (B) Purified SsaN has ATPase activity. SsaN activity was tested in a pyruvate kinase-lactate dehydrogenase coupled assay that monitors NADH oxidation coupled with ATP hydrolysis. Shown are representative data from three experiments.
(0.57 MB PDF)

Table S1 Distribution of genetic loci in the Genus *Salmonellae*. The presence (+) or absence (−) of *srcA*, SPI-1, SPI-2, *pipB2* and *sseL* in sequenced serotypes of *Salmonella enterica* and *S. bongori* are shown, along with the percent identity to SL1344 sequences in brackets.
(0.07 MB PDF)

Protocol S1 Recipe for standard minimal medium (LPM) compatible with SILAC mass spectrometry.
(0.06 MB PDF)

Dataset S1 SILAC dataset. Accession numbers correspond to protein coding sequences in *Salmonella enterica* serovar Typhimurium strain SL1344.
(0.03 MB XLS)
