# Supplementary material for: Structural and Biochemical Characterization of SrcA, a Multi-Cargo Type III Secretion Chaperone in Salmonella Required for Pathogenic Association with a Host
Source: PLoS Pathog. 2010 Feb 5;6(2):e1000751. doi: 10.1371/journal.ppat.1000751 (PMC2816692; doi:10.1371/journal.ppat.1000751)
Supplement: Figure S1 — Sequence and biochemical analysis of Salmonella SsaN. (A) Amino acid sequence alignment of SsaN and EscN from E. coli. The conserved Walker A and Walker B boxes from P-loop NTPases are shown in red and blue, respectively. Additional conserved catalytic residues are indicated in green including: Glu191 (conserved in position and orientation in EscN with F1 ATPase β-subunit); Arg352 (protrudes from adjacent monomer to bind ATP γ-phosphate in the ATP binding pocket of adjacent monomer); Gln412 (stabilizes ATP binding pocket in EscN). Residues correspond to SsaN numbering. (B) Purified SsaN has ATPase activity. SsaN activity was tested in a pyruvate kinase-lactate dehydrogenase coupled assay that monitors NADH oxidation coupled with ATP hydrolysis. Shown are representative data from three experiments. (0.57 MB PDF) [file ppat.1000751.s001.pdf]

A

```
SsaN MKNE---LMQRLRLKYP-------------PPDGYCRW-GRIQDVSATLLNAWLPGVFMG
EscN MISEHDFVLER----YPRIQRVLNSTVPTLSLNSSTRYEGKITNIGGTIIKARLPKARIG
     * .*   :::*    **              . :.  *: *:* ::..*:::* ** . :*

SsaN ELCCIKPGEELAEVVGINGSKALLSPFTSTIGLHCGQQVMALRRRHQVPVGEALLGRVID
EscN AFYRIEPSQRLAEVIAIDEDEVFLLPFEHISGMYCGQWLSYQGDEFKIRVGDELLGRLVD
      :  *:*.:.****:.*: .:.:* **    *::*** :      ..:: **: ****::*

SsaN GFGRPLDGR----ELPDVCWKDYDAMPPPAMVRQPITQPLMTGIRAIDSVATCGEGQRVG
EscN GIGRPMGSNITAPYLP--FERSLYAEPPDPLLRQVIDQPFTLGVRAIDGLLTCGIGQRIG
     *:***:...      **     :.  * ** .::** * **:   *:****.: *** ***:*
   Walker-A
SsaN IFSAPGVGKSTLLAMLCNAPDADSNVLVLIGERGREVREFIDFTLSEETRKRCVIVVATS
EscN IFAGSGVGKSTLLGMICNGASADIIVLALIGERGREVNEFLAL-LPQSTLSKCVLVVTTS
     **:..********.*:**...**  **.*********.**: : *.:.* .:**:**:**
                                 Walker-B
SsaN DRPALERVRALFVATTIAEFFRDNGKRVVLLADSLTRYARAAREIALAAGETAVSGEYPP
EscN DRPALERMKAAFTATTIAEFFRDQGKNVLLMMDSVTRYARAARDVGLASGEPDVRGGFPP
     *******::* *.**********:**.*:*: **:********::.**:**. * * :**

SsaN GVFSALPRLLERTGMGEKGSITAFYTVLVEGDDMNEPLADEVRSLLDGHIVLSRRLAERG
EscN SVFSSLPKLLERAGPAPKGSITAIYTVLLESDNVNDPIGDEVRSILDGHIVLTRELAEEN
     .***:**:****:* . ******:****:*.*::*:*:.*****:*******:*.***..

SsaN HYPAIDVLATLSRVFPVVTSHEHRQLAAILRRCLALYQEVELLIRIGEYQRGVDTDTDKA
EscN HFPAIDIGLSASRVMHNVVTSEHLRAAAECKKLIATYKNIELLIRIGEYTMGQDPEADKA
     *:****:  : ***:  *.: ** : **  :: :* *:::*********  * *.::***

SsaN IDTYPDICTFLRQSKDEVCGPELLIEKLHQILTE
EscN IKNRKAIQSFIQQSTKDISSYEKTIESLFKVVA-
     *..   * :*::**..::.. *  **.*.::::
```

B

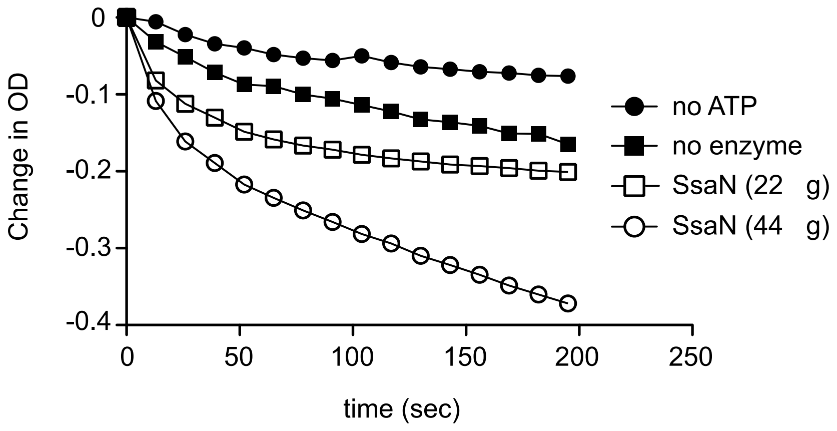

Cooper et al. Supplementary Figure 1
