# Supplementary material for: Structural and Biochemical Characterization of SrcA, a Multi-Cargo Type III Secretion Chaperone in Salmonella Required for Pathogenic Association with a Host
Source: PLoS Pathog. 2010 Feb 5;6(2):e1000751. doi: 10.1371/journal.ppat.1000751 (PMC2816692; doi:10.1371/journal.ppat.1000751)
Supplement: Table S1 — Distribution of genetic loci in the Genus Salmonellae. The presence (+) or absence (−) of srcA, SPI-1, SPI-2, pipB2 and sseL in sequenced serotypes of Salmonella enterica and S. bongori are shown, along with the percent identity to SL1344 sequences in brackets. (0.07 MB PDF) [file ppat.1000751.s002.pdf]

Table S1: Distribution of genetic loci in the Genus *Salmonellae*

Species (strain)	*srcA*[a]	SPI-1	SPI-2	*pipB2*	*sseL*
S. enterica					
Typhimurium (LT2)	+ (100)	+	+	+ (100)	+ (100)
Typhimurium (SL1344)	+ (100)	+	+	+ (100)	+ (100)
Typhi (Ty2)	+ (97)	+	+	+ (95)	+ (93)
Typhi (CT18)	+ (97)	+	+	+ (95)	+ (93)
Paratyphi A	+ (97)	+	+	+ (98)	+ (93)
Choleraesuis	+ (99)	+	+	+ (98)	+ (93)
Newport (SL254)	+ (100)	+	+	+ (98)	+ (96)
Dublin (CT02021853)	+ (99)	+	+	+ (99)	+ (93)
Heidelberg (SL476)	+ (99)	+	+	+ (99)	+ (93)
Heidelberg (SL486)	+ (99)	+	+	+ (99)	+ (93)
Agona (SL483)	+ (98)	+	+	+ (94)	+ (94)
Schwarzengrund (SL480)	+ (98)	+	+	+ (94)	+ (91)
Schwarzengrund (CVM19633)	+ (98)	+	+	+ (94)	+ (91)
Enteriditis	+ (98)	+	+	+ (99)	+ (94)
Gallinarum	+ (98)	+	+	+ (99)	+ (94)
S. bongori	-	+	-	-	-

[a] +, present; -, absent (% identity to SL1344 orthologue)
