# Supplementary material for: Structural and Biochemical Characterization of SrcA, a Multi-Cargo Type III Secretion Chaperone in Salmonella Required for Pathogenic Association with a Host
Source: PLoS Pathog. 2010 Feb 5;6(2):e1000751. doi: 10.1371/journal.ppat.1000751 (PMC2816692; doi:10.1371/journal.ppat.1000751)
Supplement: Protocol S1 — Recipe for standard minimal medium (LPM) compatible with SILAC mass spectrometry. (0.06 MB PDF) [file ppat.1000751.s003.pdf]

Protocol S1 – SILAC-LPM Medium (Cooper et al.)

For standard LPM minimal medium, the final concentration of the various components is:

5 mM KCl
7.5 mM $(NH_4)_2SO_4$
0.5 mM K_2SO_4
80 mM MES
38 mM glycerol (0.3% v/v)
Amino acids (1/100 dilution of a mixture containing the [amino acids] in 10% casaminoacids (see below)
24 μM $MgCl_2$
500 μM PO_4^{3-} (added as mixture of mono- and dibasic phosphate)

The first 4 ingredients are prepared as a 5 X salts solution and autoclaved to sterilize. To prepare the working medium, the salts solution, $MgCl_2$, phosphate ions, glycerol and casamino acids are combined. The pH is adjusted to 5.8 and the medium is filter sterilized prior to use.

For SILAC minimal medium, the casamino acid component of LPM is replaced with individual amino acids as follows:

	**% of dry cas**	**Weight (g) for 100 ml stock**	**Molarity to mock aa in 10% [0.1%] cas (mM)**
Alanine	3.0	0.30	34 [0.34]
Asparagine	0.0	0.0	0.0 [-]
Aspartic acid	2.4	0.24	18 [0.18]
Cysteine	0.1	0.01	0.83 [0.0083]
Glutamic acid	15.9	1.59	108 [1.08]
Glutamine	0.0	0.0	0.0 [-]
Glycine	1.4	0.14	19 [0.19]
Histidine	0.8	0.08	5.2 [0.052]
Isoleucine	4.0	0.40	30 [0.30]
Leucine	5.0	0.50	38 [0.38]
Methionine	1.4	0.14	9.4 [0.094]
Phenylalanine	3.6	0.36	22 [0.22]
Proline	8.0	0.80	69 [0.69]
Serine	2.1	0.21	20 [0.20]
Threonine	1.5	0.15	13 [0.13]
Tryptophan	0.0	0.0	0.0 [-]
Tyrosine	0.4	0.04	2.2 [0.022]
Valine	5.6	0.56	48 [0.48]
Arginine*	2.5	0.25	14 [0.14]
Lysine*	5.2	0.52	36 [0.36]

*omit arginine and lysine from the stock when labelling for SILAC
